# Internet Interest in Colon Cancer Following the Death of Chadwick Boseman: Infoveillance Study

**DOI:** 10.2196/27052

**Published:** 2021-06-15

**Authors:** Hiten Naik, Maximilian Desmond Dimitri Johnson, Michael Roger Johnson

**Affiliations:** 1 Department of Medicine University of British Columbia Vancouver, BC Canada; 2 Integrated Engineering University of British Columbia Vancouver, BC Canada; 3 Beedie School of Business Simon Fraser University Vancouver, BC Canada

**Keywords:** colon cancer, Google, Wikipedia, infodemiology

## Abstract

**Background:**

Compared with White Americans, Black Americans have higher colon cancer mortality rates but lower up-to-date screening rates. Chadwick Boseman was a prominent Black American actor who died of colon cancer on August 28, 2020. As announcements of celebrity diagnoses often result in increased awareness, Boseman’s death may have resulted in greater interest in colon cancer on the internet, particularly among Black Americans.

**Objective:**

This study aims to quantify the impact of Chadwick Boseman’s death on web-based search interest in colon cancer and determine whether there was an increase in interest in regions of the United States with a greater proportion of Black Americans.

**Methods:**

We conducted an infoveillance study using Google Trends (GT) and Wikipedia pageview analysis. Using an autoregressive integrated moving average algorithm, we forecasted the weekly relative search volume (RSV) for GT search topics and terms related to colon cancer that would have been expected had his death not occurred and compared it with observed RSV data. This analysis was also conducted for the number of page views on the Wikipedia page for colorectal cancer. We then delineated GT RSV data for the term *colon cancer* for states and metropolitan areas in the United States and determined how the RSV values for these regions correlated with the percentage of Black Americans in that region. Differences in these correlations before and after Boseman’s death were compared to determine whether there was a shift in the racial demographics of the individuals conducting the searches.

**Results:**

The observed RSVs for the topics *colorectal cancer* and *colon cancer screening* increased by 598% and 707%, respectively, and were on average 121% (95% CI 72%-193%) and 256% (95% CI 35%-814%) greater than expected during the first 3 months following Boseman’s death. Daily Wikipedia page view volume during the 2 months following Boseman’s death was on average 1979% (95% CI 1375%-2894%) greater than expected, and it was estimated that this represented 547,354 (95% CI 497,708-585,167) excess Wikipedia page views. Before Boseman’s death, there were negative correlations between the percentage of Black Americans living in a state or metropolitan area and the RSV for *colon cancer* in that area (*r*=−0.18 and *r*=−0.05, respectively). However, in the 2 weeks following his death, there were positive correlations between the RSV for colon cancer and the percentage of Black Americans per state and per metropolitan area (*r*=0.73 and *r*=0.33, respectively). These changes persisted for 4 months and were all statistically significant (*P*<.001).

**Conclusions:**

There was a significant increase in web-based activity related to colon cancer following Chadwick Boseman’s death, particularly in areas with a higher proportion of Black Americans. This reflects a heightened public awareness that can be leveraged to further educate the public.

## Introduction

### Background

On August 28, 2020, it was announced through Chadwick Boseman’s Twitter account that he had died of colon cancer at the age of 43 years. Boseman was a Black American actor who was perhaps best known for playing the Black Panther in the Marvel Cinematic Universe. The announcement was shocking, as he was a seemingly healthy young man whose cancer diagnosis was not disclosed to the public. Boseman is an icon in the Black community. In addition to his lauded role as the fictional Black Panther, he also played several prominent, historically impactful Black Americans in his movies, such as the baseball player Jackie Robinson, funk musician James Brown, and civil rights activist and supreme court justice Thurgood Marshall. The surprising news of his death was shared widely on social media, and the tweet soon became the most liked in Twitter history [1].

Boseman’s death was a tragic loss, but it may have had a beneficial aspect. The degree of attention that it spawned may result in a greater awareness about colon cancer, a disease that is curable if caught early by established screening methods. Prior research has demonstrated that the announcement of cancer or related diagnosis by a celebrity results in a heightened interest in that cancer and that this can extend to greater participation in screening and primary prevention [2-7]. Probably the most well-known case is the *Angelina effect*, a term coined by Time magazine [8] to describe the impact that Angelina Jolie had in 2013 when she publicly disclosed in a New York Times editorial that she had undergone a risk-reducing bilateral mastectomy after learning that she was a BRCA1 (Breast Cancer Gene 1) mutation carrier [9]. Public interest in breast cancer has soared, and there has been a subsequent increase in genetic testing [2,7]. Similarly, it is possible that Boseman’s death will lead to increased uptake of colon cancer stool tests and screening colonoscopies in the coming years.

However, it is unclear whether this spike in interest from celebrity-related news truly has a positive impact on health-related behaviors in the long term [6,10]. A 2016 analysis of the Angelina effect revealed that although the rates of breast cancer gene testing increased following her New York Times editorial, the rates of mastectomies among those tested declined [6]. This implied that the women who underwent the genetic tests after her revelation had a lower pretest probability of testing positive than those tested before. The authors concluded that celebrity announcements might not effectively target the subpopulation at the highest risk of a particular disease [6]. In contrast, the fallout of Boseman’s death has the potential to be different because he was a Black American. Despite having a greater risk of colon cancer, Black Americans lag behind White Americans with respect to up-to-date screening rates [11]. If Boseman’s legacy results in an increased awareness about colon cancer that leads to an increased screening in Black communities, the impact could be truly substantial in lowering the incidence of colon cancer.

### Research Objectives

It is likely too early to determine whether Boseman’s death has had a national effect on the rates of colon cancer screening and diagnoses. However, public interest in colon cancer can be evaluated sooner by monitoring the relative internet activity surrounding the topic. In an emerging field known as *infodemiology*, researchers have used the activity on search engines and other websites to make inferences about health issues at the population level [12-16]. This process of real-time surveillance of the supply and demand of health information has been dubbed *infoveillance* [12,13,16].

Among the most common tools for infoveillance is Google Trends (GT), a feature provided by Google that allows for quantification of Google searches for specific terms or broader topic areas [17]. For example, GT has been used to monitor outbreaks of infectious diseases [14,15,18-20], infer cancer incidences in specific geographic locations [3,21], and understand trends in health behaviors such as smoking cessation [22,23], suicide rates [24], uptake of cannabis products [25], and cancer screening [26,27]. It has also proven to be a valuable tool for tracking the impact of public health campaigns [22,28] and celebrity-related news [23,29,30]. For example, by assessing search volumes, it has been determined that there was an increased interest in condoms and HIV testing following Charlie Sheen’s disclosure of HIV [29], Asperger syndrome following Greta Thurnberg appearances [31], and mental health hotlines following Demi Lovato’s overdose and Anthony Bourdain’s suicide [30].

Although not as widely used as GT data, quantifying the number of page views of disease-specific articles on Wikipedia has also been used to evaluate public interest in that disease. Studies have documented increases in page views in temporal relation to celebrity diagnosis and deaths. For example, a study showed that there were more page views on the Wikipedia article for strokes after Margaret Thatcher’s death, on the multiple sclerosis article when Ozzy Osbourne announced his diagnosis, and on the epilepsy article when Lil Wayne was hospitalized with seizures [32]. Similarly, there was an increase in traffic on the Wikipedia article for vasculitis soon after Harold Allen Ramis died of that condition [33].

What remains under study is how this infoveillance data can be effectively leveraged to improve health outcomes for the public. In the examples cited, there is typically a spike in activity following the event in question before returning to baseline [29,31,33]. An important objective would be for policy makers to use these data to address disparities in diseases such as colon cancer, and to accomplish this, it would be valuable to understand patterns of interest for particular demographic groups such as in Black Americans. Although it is not possible to know the identities of the individuals who have elevated interest, GT allows the search volume data to be granulated down to specific geographic areas. Understanding the demographics of the areas with heightened activity following an event can help us infer which demographic groups had increased interest so that they may be targeted in public health campaigns featuring the celebrity.

Therefore, characterizing the impact of Chadwick Boseman’s death on the internet would be valuable to public health leaders and colon cancer advocacy groups who aim to address racial disparities. The goals of this study are twofold. First, we use GT and Wikipedia page view data to evaluate the degree of increased interest in colon cancer and related topics and terms following Boseman’s death. Second, we analyze the geographic distribution of this increased interest to determine if it disproportionately originated from Black Americans. With this information, we seek to provide a framework that could be used to develop targeted campaigns aimed at increasing screening and awareness of colon cancer, particularly among Black people in the United States.

## Methods

### Google Searches and Wikipedia Page Views for Colon Cancer

We used GT [34] to assess the weekly relative search volume (RSV) of topics and terms related to colon cancer in the United States. Search *terms* are specific words or phrases, whereas *topics* encompass many terms, as defined by Google, that share the same concept. The topics used were *colorectal cancer* and *colon cancer screening*. A total of 15 terms related to colon cancer were specifically chosen to represent queries related to colon cancer screening and diagnosis (*colonoscopy*, *stool test*, and *diagnosis*), symptoms and signs (*stool*, *symptoms*, *signs*, and *anemia*), risk factors (*risk*, *men*, *age*, *black (-panther)*, and *African American*), treatment (*treatment*), and prognosis (*survival*, *death*). According to Google, RSV is calculated by dividing the number of searches for a particular term or topic by the total searches based on geography and time range. The resulting numbers are then scaled from 0 to 100 to provide a relative indication of the popularity of the search query. For each topic and term, weekly RSV data points were downloaded for the United States for a period of 2 years before Boseman’s death to 3 months after (week of September 2, 2018, to week of November 29, 2020). Each topic and term was used as an independent search query for GT.

We also used page views analysis [35] to extract data regarding the number of views of the English Wikipedia page for colorectal cancer between 2 years before Boseman’s death and 3 months after (August 28, 2018, to November 29, 2020). The daily views of this page were then normalized using the total number of views of all English Wikipedia pages for each day.

On the basis of the historical data 2 years before Boseman’s death, we conducted quasi-experimental analyses by forecasting the weekly Google RSV and the daily number of Wikipedia page views that would be expected if his death had not occurred and compared it with the observed values. The forecasts were generated with 95% bootstrapped CIs using an advanced autoregressive integrated moving average algorithm in the forecast package [36] of the R software (version 4.0.3; R Foundation).

### Geographic Patterns in Searches for Colon Cancer

To investigate a possible shift in demographics relative to search volume for colon cancer, we analyzed Google RSV data further characterized by state and metropolitan regions. The *Interest in subregion* option on GT generates RSV values for each specific state for a search term during a given period. The RSV is scaled from 0-100 based on the popularity relative to the total number of Google searches performed during a specified time in the specified states where there is sufficient data. Instead of by state, this can be done separately by metropolitan regions in the United States, which on GT is delineated based on Nielsen designated market area (DMA) boundaries. We used the search term *colon cancer* and extracted RSV data for the period of 2 years before Boseman’s death (August 28, 2018, to August 27, 2020) and periods up to 4 months following his death (August 28, 2020 to December 28, 2020).

We then compared these RSV values with the percentages of Black, White, and Hispanic and Latino American populations for each of the states and metropolitan areas and conducted correlation analyses. These correlations were expressed as the Pearson coefficient of correlation (*r*), and correlations were compared between the period 2 years before Boseman’s death and time intervals up to 4 months following his death. This comparison was performed using a statistical test to compare 2 overlapping correlations based on dependent groups [37]. Visualizations (scatter plots and maps) were created using Tableau Desktop (Tableau Software LLC). For mapping purposes, each state or metropolitan region was shaded based on how the RSV values differed from the mean RSV value of all state or metropolitan regions during that period to highlight relative increases and decreases before and after Boseman’s death.

Demographic information regarding the percentage of each race in each state and DMA was derived from the 2019 US Census data. State-level data were extracted from the United States Census Bureau website [38]. Data regarding the percentage of each race in each DMA were purchased from Cubit Planning Inc (Austin) through a custom data request. This company had previously developed a strategy to derive this information by extracting county-specific demographic data from US Census data sets and then determining which county or counties comprised each DMA [39].

## Results

### Google Searches for Colon Cancer

**Figure 1:**
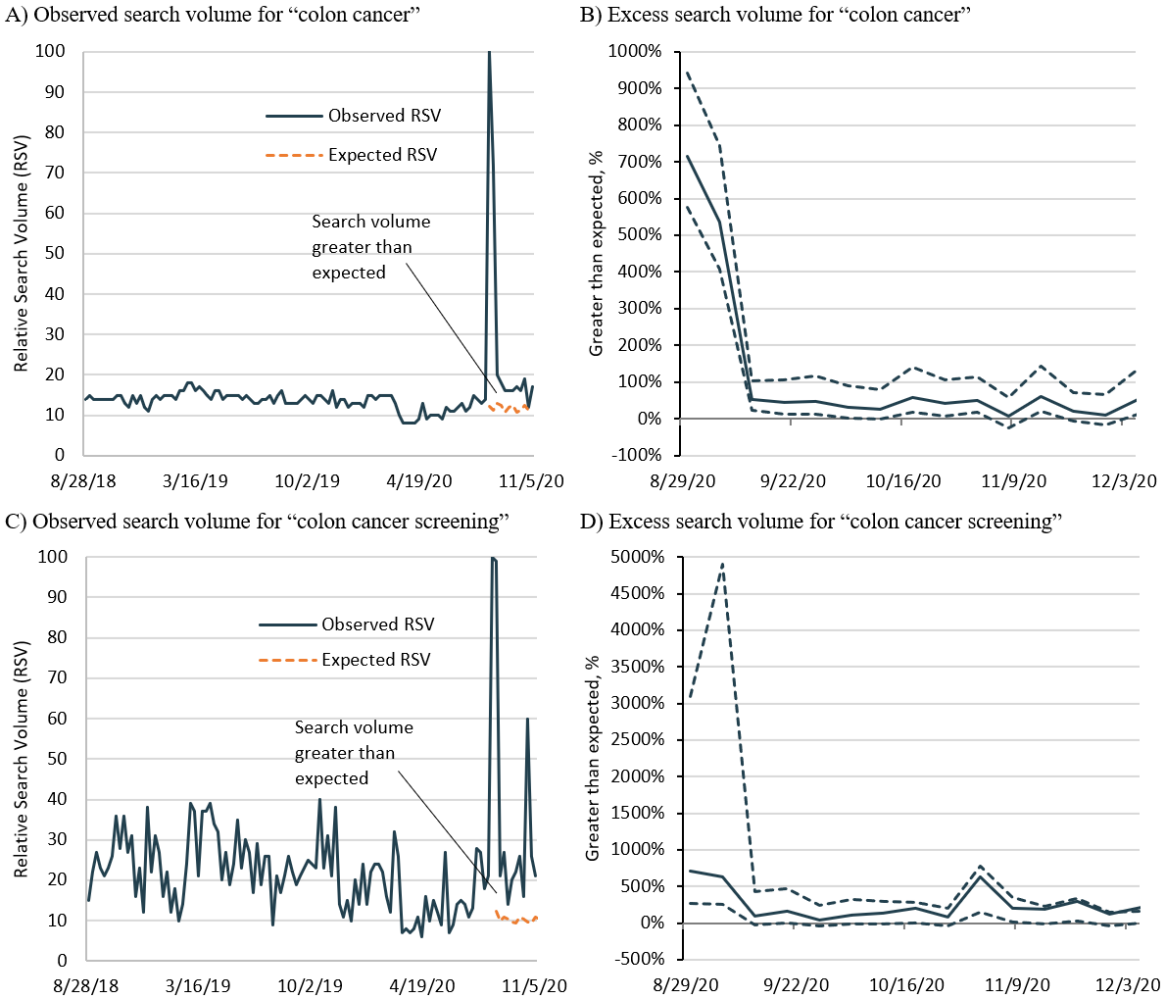
Greater than expected relative search volumes for colon cancer following Chadwick Boseman’s death. RSV: relative search volume.

Following August 28, 2020, GT observed RSV values for the search term *colon cancer* and the search topic *colon cancer screening* increased beyond the expected RSV by 716% and 707%, respectively (Figure 1). In the case of the search term *colon cancer*, observed RSV values remained significantly greater than expected for at least 2 months, during which searches were on average 189% (95% CI 132%-291%) greater than expected. By the third month, the average observed RSV was 118% (95% CI 72%-201%) greater than expected. Searches for the topic *colon cancer screening* were also significant 2 months following August 28, 2020, with an average of 261% (95% CI 54%-1252%) greater than expected (Figure 1). Similarly, by the third month, the average observed RSV remained significantly greater than expected, with an average of 256% (95% CI 35%-814%).

**Figure 2:**
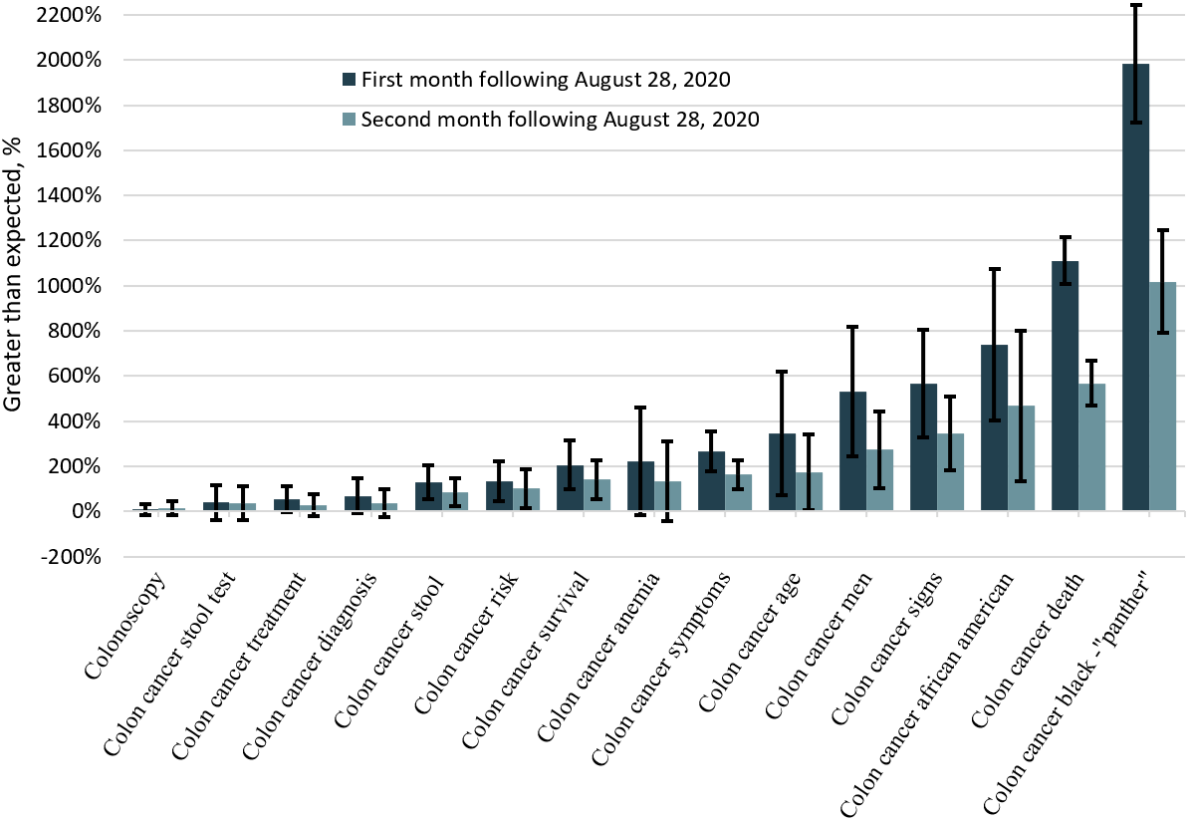
Greater than expected relative search volumes for Google search terms related to colon cancer following Chadwick Boseman’s death.

When analyzing related GT search terms for colon cancer, 10 of 15 had mean observed RSVs significantly higher than expected within the first 2 months (Figure 2), and of these, 3 (*colon cancer signs*, *colon cancer survival*, and *colon cancer symptoms*) remained significantly greater than expected within the third month.

### Wikipedia Page Views for Colon Cancer

**Figure 3:**
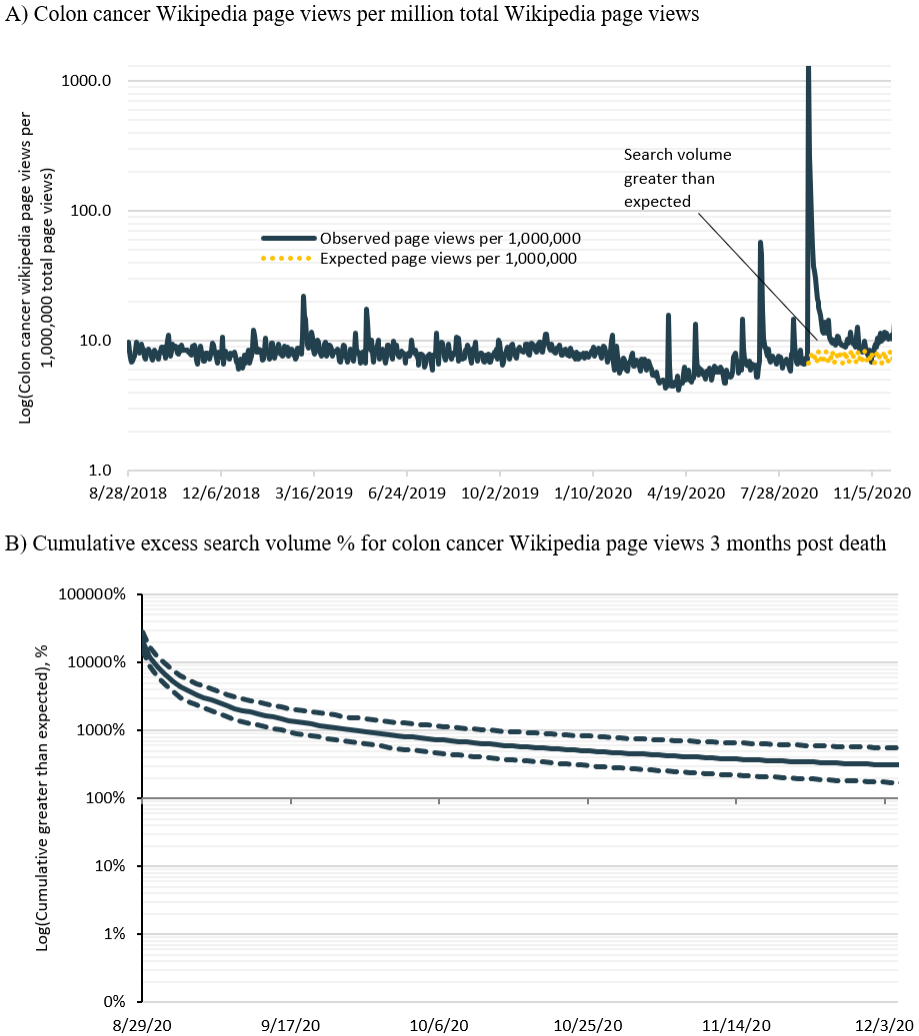
Excess page views for the Wikipedia page colorectal cancer following Chadwick Boseman’s death.

Daily page views for the English Wikipedia page *colorectal cancer* peaked at 1386.7 per million total views or 20,649% (95% CI 15,188%-27,901%) greater than expected on August 29 before declining (Figure 3). As shown in Figure 3, the cumulative excess search volume during this period was on average 1979% (95% CI 1375%-2894%) greater than expected. Altogether, it was estimated that there were 547,354 (95% CI 49,7708-58,5167) excess Wikipedia page views during the 2 months following the news of Boseman’s death.

### Geographic Patterns in Searches for Colon Cancer

**Figure 4:**
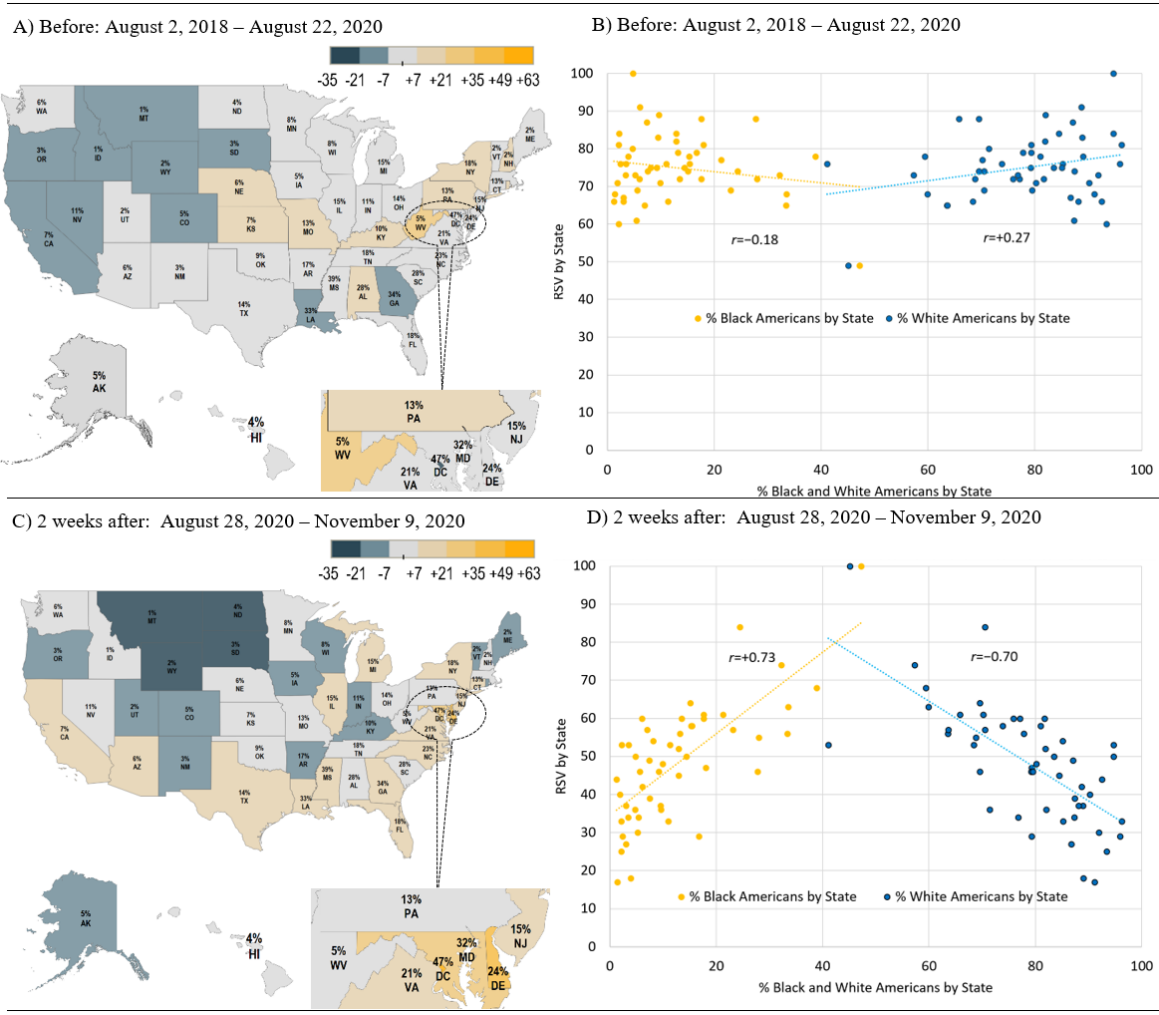
Shifts in demographics and Google relative search volumes for Colon Cancer by state before and after Chadwick Boseman’s death. The percentage of the Black American population by state is indicated in a smaller text on the map based on the 2019 US Census. The shading scale is based on the difference between the relative search volume value for each state and the mean relative search volume for all states during the specified period.

As shown in Figure 4, for 2 years before August 28, 2020, RSV for the search term *colon cancer* was greatest among states in the northeast extending through the Midwest to Nebraska (with Alabama being the only southern state above the average RSV). In the 2 weeks following August 28, there was a geographic shift, such that states in the south tended to have more search interest. The lists of the states that had the most search interest and change in search interest following Boseman’s death are outlined in Multimedia Appendix 1, Table S1. The percentage of the Black American population in each state as per the 2019 US Census is indicated in Figure 4, and for each DMA in Multimedia Appendix 1, Figure S1.

**Figure 5:**
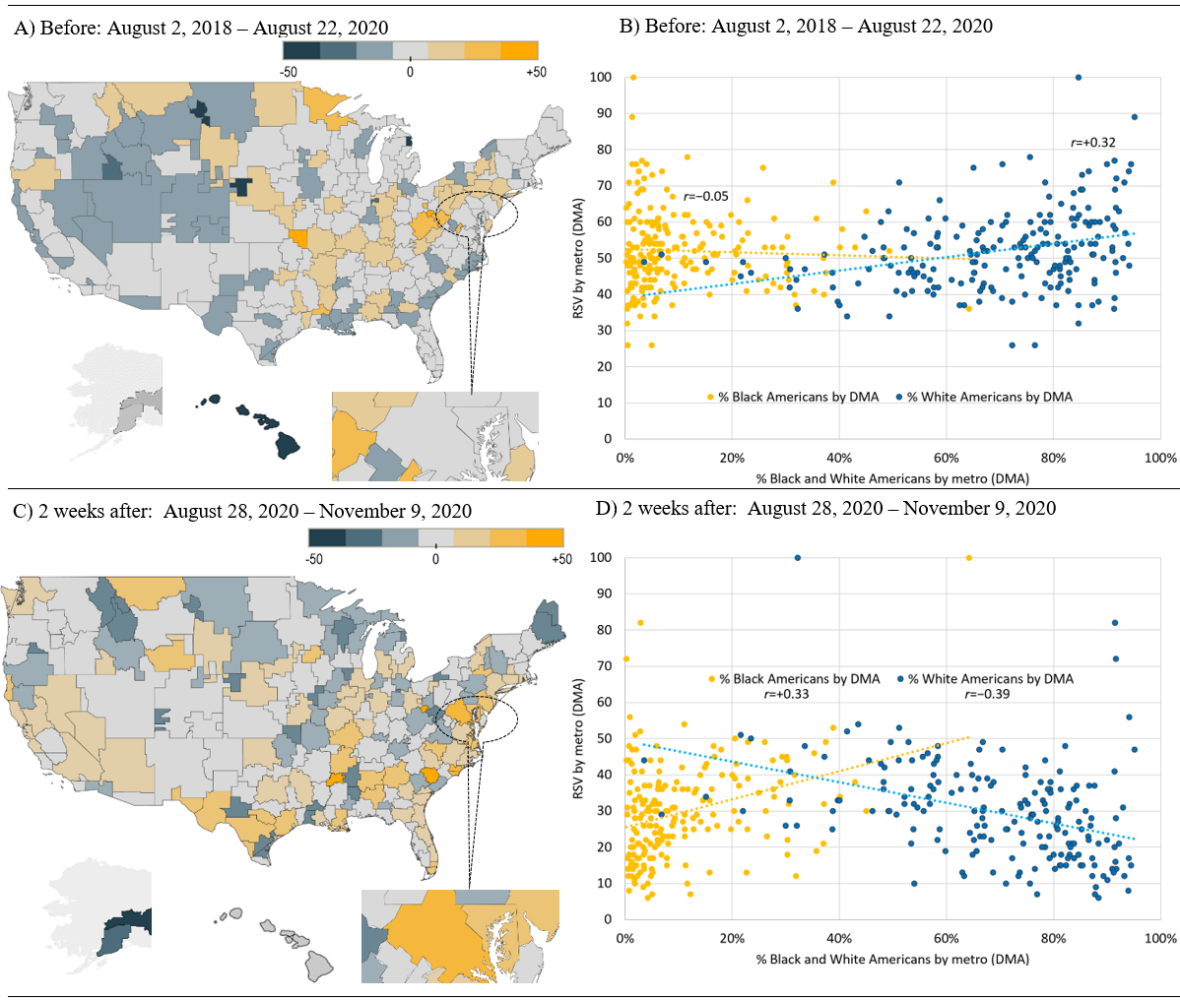
Shifts in demographics and Google relative search volumes for colon cancer by metropolitan area before and after Chadwick Boseman’s death. Metropolitan areas are delineated based on Nielsen designated market areas. The shading scale is based on the difference between the relative search volume value for each metropolitan area and the mean relative search volume for all states during the specified period. DMA: designated market area.

A similar geographic shift was observed when examining metropolitan areas defined using the DMA boundaries (Figure 5). To determine how this shift corresponded to changes in interest among racial groups, we conducted a series of correlation analyses. As shown in Figures 4 and 5, before August 28, 2020, there was a slight negative correlation between the RSV for colon cancer and the percentage of Black Americans per state and per metropolitan area (*r*=−0.18 and *r*=−0.05, respectively) and a slight positive relationship for the percentage of White Americans per state and per metropolitan area (*r*=0.27 and *r*=0.32, respectively). In the 2 weeks following August 28, 2020, there was a significant shift in correlation within racial demographics and RSV for *colon cancer* by state and metropolitan area, such that there was a positive correlation between the RSV for colon cancer and the percentage of Black Americans per state (*r*=0.73 and *r*=0.33, respectively) and a negative relationship for the percentage of White Americans per state and metropolitan area (*r*=−0.70 and *r*=−0.39, respectively). These changes in correlations were all highly significant (*P*<.001), relative to the corresponding correlations during the 2 years before August 28, 2020.

This analysis was repeated for periods extending up to 4 months following Boseman’s death. As shown in Table 1, after August 28, 2020, the correlations remain positive between the RSV for *colon cancer* and the percentage of Black Americans by state and metropolitan area. Similarly, the percentage of White Americans and the RSV for colon cancer is negative and significantly lower after August 28, 2020, for all periods compared by state and metropolitan area. As shown in Table 1, Hispanic and Latino Americans have a similar pattern as Black Americans concerning RSV for colon cancer with time.

**Table 1:** Correlations between search interest in colon cancer and racial demographics.

Search terms and racial demographics				Google search dates^a^												
				Before (September 2, 2018-August 22, 2020)		1 week after (August 28, 2020-September 4, 2020)		2 weeks after (August 28, 2020-September 11, 2020)		4 weeks after (August 28, 2020-September 25, 2020)		8 weeks after (August 28, 2020-October 23, 2020)		12 weeks after (August 28, 2020-November 20, 2020)		4 months after (August 28, 2020-December 31, 2020)
***Colon cancer* by state**																
	**Black Americans**															
		Correlation coefficient, *r*	−0.18		0.51		0.73		0.68		0.62		0.69		0.65	
		*P* value	—^b^		<.001		<.001		<.001		<.001		<.001		<.001	
	**White Americans**															
		Correlation coefficient, *r*	0.27		−0.69		−0.70		−0.68		−0.69		−0.70		−0.46	
		*P* value	—		<.001		<.001		<.001		<.001		<.001		<.001	
	**Hispanic and Latino Americans**															
		Correlation coefficient, *r*	−0.27		0.21		0.18		0.20		0.20		0.12		0.11	
		*P* value	—		.03		.04		.03		.03		—		—	
***Colon cancer* by metropolitan area (DMA^c^)**																
	**Black Americans**															
		Correlation coefficient, *r*	−0.05		0.24		0.32		0.20		0.28		0.20		0.25	
		*P* value	—		.004		<.001		.002		<.001		<.001		<.001	
	**White Americans**															
		Correlation coefficient, *r*	0.32		−0.27		−0.38		−0.35		−0.34		−0.31		−0.34	
		*P* value	—		<.001		<.001		<.001		<.001		<.001		<.001	
	**Hispanic and Latino Americans**															
		Correlation coefficient, *r*	−0.29		0.12		0.22		0.23		0.16		0.18		0.18	
		*P* value	—		<.001		<.001		<.001		<.001		<.001		<.001	

^a^Each value represents the Pearson correlation coefficient between the relative search volume and the percentage of the population for each race in the corresponding jurisdiction.

^b^N/A: not applicable.

^c^DMA: designated market area (Google Trends defines metro areas according to 210 DMAs).

## Discussion

### Principal Findings and Implications

In this infoveillance study, we examined the impact of Chadwick Boseman’s death from colon cancer on the interest in this disease on the internet. We found that Boseman’s death resulted in a dramatic and significant increase in Google searches related to colon cancer as well as in the number of page views of the colon cancer Wikipedia article. We further investigated how the increased Google search interest in colon cancer was distributed geographically by state and metropolitan areas. Our findings demonstrated a significant shift in correlation within the Black American population and RSV for colon cancer in state and metropolitan regions. This correlation remained for at least 4 months afterward.

Research studies thus far have shown that searches for colonoscopy have increased with time [27] and correlate with actual screening rates in the United States [26]. However, although current campaigns such as the colon cancer awareness month result in increased interest on the web, these campaigns do not seem to generate enough impact to result in increased screening [40]. Involving the stories of celebrities like Chadwick Boseman in these campaigns may be a more effective strategy. Previously, the disclosure of cancer diagnoses from other celebrities has resulted in higher screening rates. For example, when Kylie Minogue announced her breast cancer diagnosis in 2005, there was an uptick in mammography appointments in Australia [5]. Similarly, there was a temporary increase in pap smear rates in the United Kingdom after Jade Goody was diagnosed with cervical cancer in 2008 [4]. It is possible that Boseman’s death will have a comparable, if not greater, impact on colon cancer screening rates.

Although analyzing screening and corresponding detection rates of cancers takes time, infodemiology research can provide early insights that public health officials can act upon. Multiple studies have examined the changes in interest on the web concerning a celebrity event, and several have used a quasi-experimental approach like ours, in which increased search volumes following an event are quantified in relation to counterfactual forecasted volumes if the event had not occurred [23,30,33]. For example, studies have reported that search queries regarding smoking cessation interest in Brazil increased by up to 153% in the first week after president Lula da Silva’s diagnosis of laryngeal cancer [23], HIV search queries in the United States increased by 417% in the week after Charlie Sheen’s disclosure [29], and there was a 257% increase in vasculitis-related queries in the United States after Harold Allen Ramis’ death [33]. In contrast, our study determined that the magnitude of increased search volume attributed to the *Chadwick Boseman effect* was found to be higher, with search queries related to colon cancer screening increasing by up to 707%.

This greater volume of web-based interest in colon cancer could be attributed to Boseman’s widespread popularity and the sheer surprise of his death. However, it is likely that social media played an important role as well, as unlike most other celebrity examples, Boseman’s diagnosis was shared via Twitter [1]. This original tweet quickly went viral, and the news spread on multiple platforms. Our study showed that this increased social media activity translated to a greater appetite for knowledge of colon cancer itself, highlighting the value of platforms such as Twitter for public health campaigns. Several studies have demonstrated that social media–based campaigns can be successful in educating the public and increasing participation in cancer screening [41]. However, most of the campaigns studied thus far have focused on breast and prostate cancer and have been less effective at reaching racial minorities [41]. The Chadwick Boseman effect phenomenon demonstrated that the involvement of a highly followed celebrity might represent an effective strategy to expand the reach of these campaigns on the internet. Furthermore, if the celebrity represents a racial minority group like Boseman, it may facilitate the engagement of these particular groups.

Our analysis showed that there was proportionately more search volume for colon cancer in states and metropolitan areas that had proportionately more Black Americans. This reinforces how popular Boseman was in the Black American community and how his legacy could be used to help spread awareness of colon cancer among his fans. This geographic component of GT infoveillance has been leveraged in studies examining disease incidence [3,19]; however, its role in research examining public health behavior and disparities is less established. Our study adds to the infoveillance field by demonstrating that internet search trend data can not only be used to identify an increased public interest in a health-related topic but can also determine *where* that interest comes from and perhaps even from *whom*.

As we emerge from the COVID-19 pandemic, an important public health goal will be to re-engage the public in cancer screening. Research has revealed that cancer screening rates are lower and that cancers are being diagnosed at more advanced stages than that observed prepandemic [42]. In the United States, Black Americans have long had lower up-to-date screening rates for colon cancer [11], and this disparity may have become more pronounced during the pandemic. Therefore, it will be even more important to appeal to Black Americans in campaigns focused on colon cancer education and the importance of screening.

Although there was a substantial increase in internet activity regarding colon cancer following Boseman’s death, the activity ultimately declined, which may have been a missed opportunity to educate the public. However, by invoking Boseman’s legacy and involving living Black American celebrities in colon cancer campaigns, it may be possible to regenerate this spike in interest among Americans. This can be done on the internet but through other means, such as television commercials and the distribution of stool testing kits at the grassroots level. The GT analyses presented in our study provide a geographic framework for the jurisdictions to target in these campaigns. For example, if a public health official in the US were to start a television campaign invoking Boseman’s legacy, they may wish to target regions such as Georgia and Washington DC, where there was a higher concentration of Black Americans as well as the greatest interest in colon cancer following his death.

### Limitations

There are limitations of our study that are important to highlight. Internet activity for a particular condition is ultimately just a surrogate marker for the public interest. It is unknown how many different people performed the searches and who they were. Our geographic analyses suggested an increased interest in colon cancer in regions of the United States with more Black Americans. This does not provide direct evidence that more Black Americans were looking up information about colon cancer, and there may have been factors other than race that drove interest in these regions.

It is also unknown whether individuals who searched for information about colon cancer gleaned any valuable knowledge from their internet visits that would influence their future behavior. Although the Wikipedia page for colorectal cancer provides a variety of information, we were unable to capture how much of the material was actually reviewed by visitors. Furthermore, we also acknowledge that the search terms investigated may not represent all of the most common search strings used by the public.

### Conclusions

This infoveillance study revealed a substantial increase in Google searches and Wikipedia page views related to colon cancer after the Black American actor Chadwick Boseman died of the disease in August 2020. A geographic analysis of states and metropolitan areas in the United States demonstrated that the surge in interest might have disproportionately come from Black Americans, a group that has a higher risk of colon cancer but lower screening rates than White Americans. Further research will be required to determine whether the temporary surge in interest following Boseman’s death will translate to sustained awareness and screening rates for colon cancer.

Health care providers and public health officials can capitalize on these findings by developing awareness campaigns incorporating Boseman’s legacy and involving other Black American celebrities. Although he quietly suffered from cancer himself, Boseman generously donated to multiple charities, including those supporting underprivileged Black Americans and youth affected by cancer. In his life, Boseman was known for portraying fictional and real-life Black heroes on the movie screen, but his status as a hero himself may be further cemented in the years to come.

## Appendix

Multimedia Appendix 1 States with the highest internet search interest in "colon cancer" and the percentage of Black Americans by metropolitan area.

## Abbreviations

BRCA1: breast cancer gene 1
DMA: designated market area
GT: Google Trends
RSV: relative search volume
