# Supplementary material for: Internet Interest in Colon Cancer Following the Death of Chadwick Boseman: Infoveillance Study
Source: J Med Internet Res. 2021 Jun 15;23(6):e27052. doi: 10.2196/27052 (PMC8277405; doi:10.2196/27052)
Supplement: Multimedia Appendix 1 [file jmir_v23i6e27052_app1.docx]

Multimedia Appendix 1.

Table S1: States with the highest search interest in “colon cancer” and greatest change in interest after Chadwick Boseman’s death

| Rank | Highest RSV above mean during the period 4 months following Boseman’s death | Greatest change in RSV from prior to Boseman’s death to 4 months after |
| --- | --- | --- |
| 1 | **District of Columbia (333,312)** | **District of Columbia (333,312)** |
| 2 | **Maryland (1,946,932)** | **Georgia (3,556,697)** |
| 3 | **Georgia (3,556,697)** | Nevada (347,489) |
| 4 | **New York (3,424,002)** | **South Carolina (1,441,530)** |
| 5 | Pennsylvania (1,649,737) | **Maryland (1,946,932)** |
| 6 | Alabama (1,364,474) | **Texas (3,908,287)** |
| 7 | Illinois (1,951,681) | Florida (3,772,874) |
| 8 | Florida (3,772,874) | North Carolina (2,424,132) |
| 9 | **South Carolina (1,441,530)** | Louisiana (1,554,297) |
| 10 | **Texas (3,908,287)** | **New York (3,424,002)** |

The Black American population is indicated in parenthesis and is based on the 2019 US Census. Bolded states are those states present on both lists.

Figure S1: Percentage of Black Americans by metropolitan area as per the 2019 US Census


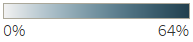

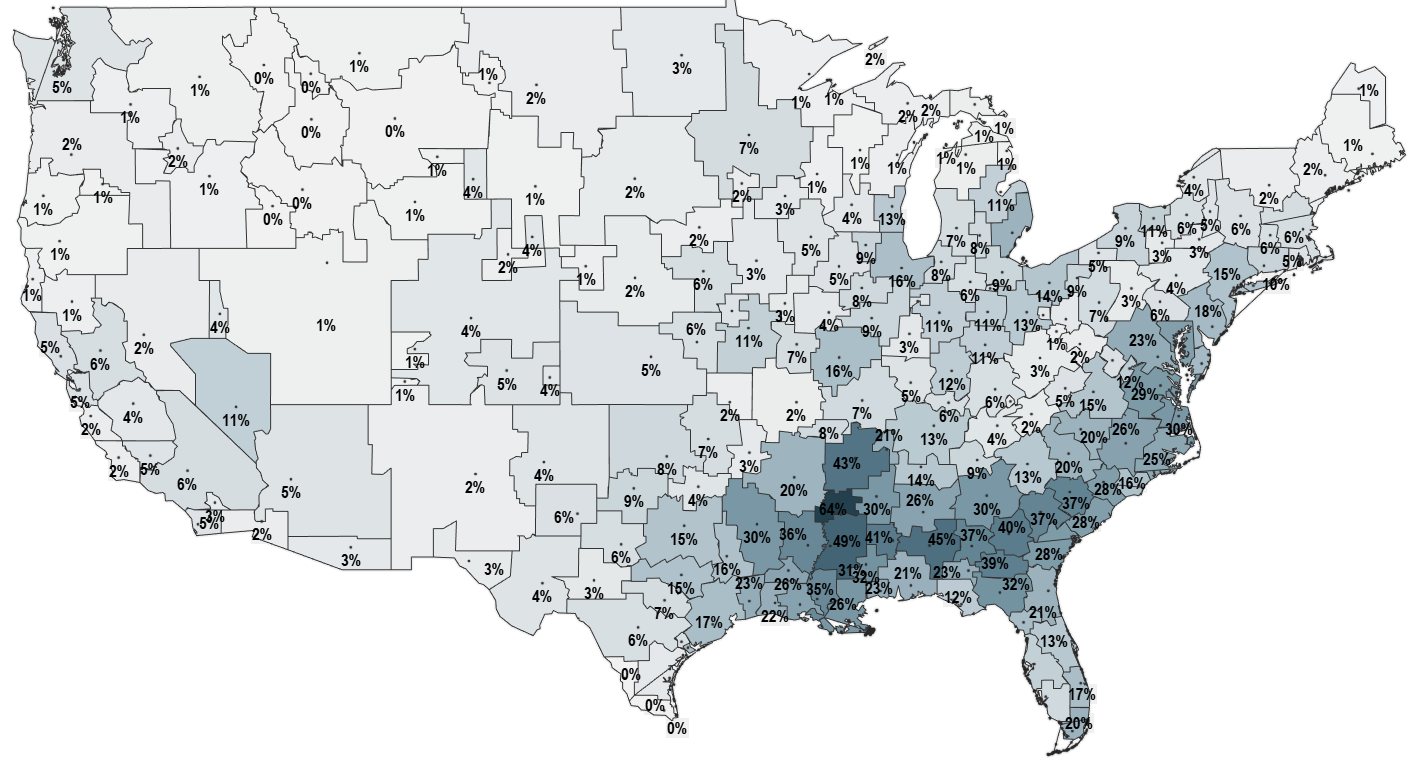


Metropolitan areas are delineated based on Neilsen’s designated market areas (DMA).
